# *Plasmodium falciparum* Gametocyte Carriage Is Associated with Subsequent *Plasmodium vivax* Relapse after Treatment

**DOI:** 10.1371/journal.pone.0018716

**Published:** 2011-04-20

**Authors:** Jessica T. Lin, Delia Bethell, Stuart D. Tyner, Chanthap Lon, Naman K. Shah, David L. Saunders, Sabaithip Sriwichai, Phisit Khemawoot, Worachet Kuntawunggin, Bryan L. Smith, Harald Noedl, Kurt Schaecher, Duong Socheat, Youry Se, Steven R. Meshnick, Mark M. Fukuda

**Affiliations:** 1 Armed Forces Research Institute of Medical Sciences, Bangkok, Thailand; 2 National Center for Parasitology, Entomology and Malaria Control, Phnom Penh, Cambodia; 3 Department of Specific Prophylaxis and Tropical Medicine, Medical University of Vienna, Vienna, Austria; 4 Department of Epidemiology, Gillings School of Public Health, University of North Carolina, Chapel Hill, North Carolina, United States of America; 5 Division of Infectious Diseases, University of North Carolina School of Medicine, Chapel Hill, North Carolina, United States of America; 6 Division of Global Emerging Infectious Disease Operations, Armed Forces Health Surveillance Center, Silver Spring, Maryland, United States of America; Université Pierre et Marie Curie, France

## Abstract

Mixed *P. falciparum*/*P. vivax* infections are common in southeast Asia. When patients with *P. falciparum* malaria are treated and followed for several weeks, a significant proportion will develop *P. vivax* malaria. In a combined analysis of 243 patients recruited to two malaria treatment trials in western Cambodia, 20/43 (47%) of those with *P. falciparum* gametocytes on admission developed *P. vivax* malaria by Day 28 of follow-up. The presence of Pf gametocytes on an initial blood smear was associated with a 3.5-fold greater rate of vivax parasitemia post-treatment (IRR = 3.5, 95% CI 2.0–6.0, p<0.001). The increased rate of post-treatment *P. vivax* infection persisted when correlates of exposure and immunity such as a history of malaria, male gender, and age were controlled for (IRR = 3.0, 95% CI 1.9–4.7, p<0.001). Polymerase chain reaction (PCR) confirmed that only a low proportion of subjects (5/55 or 9.1%) who developed vivax during follow-up had detectable Pv parasites in the peripheral blood at baseline. Molecular detection of falciparum gametocytes by reverse transcriptase PCR in a subset of patients strengthened the observed association, while PCR detection of Pv parasitemia at follow-up was similar to microscopy results. These findings suggest that the majority of vivax infections arising after treatment of falciparum malaria originate from relapsing liver-stage parasites. In settings such as western Cambodia, the presence of both sexual and asexual forms of *P. falciparum* on blood smear at presentation with acute falciparum malaria serves as a marker for possible occult *P. vivax* coinfection and subsequent relapse. These patients may benefit from empiric treatment with an 8-aminoquinolone such as primaquine.

## Introduction

Despite prevalence of both *P. falciparum* and *P. vivax* in South and Southeast Asia, Oceania, and parts of South America, mixed infections of the two species were rarely reported in the past, with cross-sectional prevalence rates reported at <5% [1]. The application of PCR detection of different malaria species has revealed that mixed infections are actually quite common and often simply go unrecognized in the field. In Thailand, it is estimated that 25–50% of malaria infections are mixed *P. falciparum/P. vivax* [1]–[3]. This estimate is based not only on PCR detection methods, but also on observations from longitudinal treatment trials conducted in the 1980s in which approximately one-third of patients treated for apparent *P. falciparum* mono-infection developed *P. vivax* infection within 28 days of treatment with short half-life antimalarials such as artesunate [4], [5]. Since these patients were removed from malaria endemic areas at enrollment, development of *P. vivax* malaria post-treatment suggested a relapse from a previously unappreciated vivax infection rather than new infection.

Subsequent antimalarial trials conducted in Southeast Asia and Papua New Guinea have continued to demonstrate high rates of *P. vivax* infection post-treatment, especially when follow-up is continued beyond the period when the antimalarials used are expected to maintain efficacious drug levels in the bloodstream [6], [7]. Recently, a study involving 811 patients in Myanmar found that 35% of those with *P. falciparum* monoinfection by peripheral smear developed *P. vivax* malaria during the 63-day follow-up period [8]. On the Thai-Burmese border, a retrospective analysis of 15 years of clinical trial data found that the cumulative 63-day risk of vivax malaria after Pf mono-infection was 51% following treatment with rapidly eliminated drugs [9]. These high rates of coinfection have led some to advocate for presumptive treatment of liver-stage vivax infection with a full course of primaquine (anti-hypnozoite therapy) in *all* patients with microscopically confirmed malaria where both species are endemic [10]. Identifying risk factors for developing relapse from a liver-stage vivax infection could help refine such a strategy. We conducted a retrospective analysis of two malaria treatment trials of uncomplicated falciparum malaria to examine whether falciparum gametocyte carriage at presentation is associated with subsequent *P. vivax* relapse.

## Methods

### Clinical studies

Between 2006–2009, two open-label clinical trials were conducted at the same site in Tasanh, western Cambodia to investigate reports of emerging artemisinin resistance. In ARC1, patients with uncomplicated *P. falciparum* malaria were randomized in a 2∶1 ratio to receive 7 days of artesunate (4 mg/kg/day) or quinine (30 mg/kg/day) plus tetracycline (25 mg/kg/day) for 7 days. In ARC2, patients with uncomplicated falciparum malaria were randomized to receive one of three artesunate dosing regimens: 2, 4, or 6 mg/kg/day for 7 days. In both studies, enrollment was limited to otherwise healthy adults with *P. falciparum* mono-infection as determined by light microscopy and with no history of antimalarial use in the 30 days prior to enrollment. The only difference in inclusion criteria between the two studies was a minimum parasite density of 1,000 parasites/µl in ARC2 compared to 100 parasites/µl in ARC1. Written informed consent was obtained from all study participants prior to enrollment. Subjects received directly observed therapy over 7 days with thick and thin blood smears prepared every 12 hours in ARC1 and every 2–6 hours in ARC2 until parasite clearance was achieved. Both studies recorded temperature every 4 hours, with fever clearance defined as the first time the patient became afebrile with absence of fever for the next 24 hours. In ARC1, patients remained in a study ward for 21 days to prevent reinfection, with weekly clinical and blood smear assessments on days 7, 14 and 21, and then returned for one final outpatient follow-up on Day 28. In ARC2, subjects remained on the study ward for the 7 days of therapy, then returned weekly until Day 42 for outpatient follow-up. Further details of these studies have been previously published [11], [12].

### Identification of parasite species

Giemsa-stained slides were examined by two microscopists blinded to each other's results and to the treatment status of the study subject. Counts were reported as the number of asexual parasites and gametocytes per 200 white blood cells seen on the thick smear. If the thick film exceeded 500 parasites per 200 WBCs, parasites were counted per 2000 RBCs (ARC1) or per 5000 RBCs (ARC2). At least 200 oil immersion fields were examined on the thick film before a blood smear was considered negative. The final count was determined by taking the geometric mean of the two microscopists' counts. Discordant results were resolved by a third reference microscopist.

Molecular identification of parasite species was performed on all admission blood samples (Day 0) from both studies and on weekly blood samples available in the ARC2 patients to assess for subpatent mixed infection at baseline and occult parasitemia during follow-up. This was done using real-time PCR assays targeting the 18srRNA gene (Schaecher et al. manuscript in preparation). Briefly, DNA extracted from 200 µLs of EDTA blood using the QIAamp® DNA blood mini kit was diluted down to a working concentration of 3 ng/µl. Primers and FAM-labeled MGB probes previously adapted from published protocols were used for a pan-species Plasmodium assay, a falciparum-specific assay, and a vivax-specific assay [13]. For each real-time PCR assay, 6.25 µl of template DNA was added to a reaction mixture containing 200 nM primers and 100 nM probe for a total reaction volume of 25 µl. Thus DNA from approximately 1 µl of blood was assayed in each PCR tube. Cycling conditions for the Applied Biosystems 7900 system were 50°C for 1 min, 95°C for 10 min, and 45 cycles of 95°C for 15 s and 60°C or 59°C for 1 min. All samples were run in triplicate with positive and negative controls in each reaction plate. A C_t_ value of 37 cycles was used as the threshold cutoff for positivity in the Pv assay. Using this C_t_ value, it was previously determined that the assay has a limit of detection for *P. vivax* of 0.5–5 parasites/µl.

### Molecular detection of gametocytes

Reverse transcriptase PCR (RT-PCR) based on the mature gametocyte marker Pfs25 was used to assess for subpatent carriage of falciparum gametocytes in admission blood samples from the patients enrolled in ARC2. RNA purified from 2.5 mLs of whole blood using the Qiagen PAXgene Blood RNA kit was diluted down to a working concentration of 50 ng/µl. cDNA synthesis and nested PCR were then carried out with the Qiagen Omniscript® RT kit and Qiagen HotStarTaq® PCR kit and primers following a previously published protocol [14]. Each RNA sample was run with an RT-minus control to assess for DNA contamination. Nested PCR runs contained a 3D7 gDNA positive control as well as a negative control. PCR products were resolved by electrophoresis on a 1.5% agarose gel, and the presence or absence of an approximately 650 bp band representing amplification of the Pfs25 transcript was determined using ultraviolet illumination.

### Statistical analysis

Microscopic and clinical data from the two studies were combined and retrospectively analyzed to determine *P. vivax* infection rates and risk factors for the development of post-treatment vivax malaria. Data was entered into Microsoft Excel and analyzed with STATA 10.1 (College Station, Texas) and SPSS 18 (Somers, NY). Clinical characteristics of the patients enrolled in ARC1 vs. ARC2 as well as those with and without gametocytes on admission were compared using Pearson Chi-Square, Mann-Whitney U, Fisher's exact test, or student's t-tests as appropriate. Kaplan Meier analysis was used to visually compare the incidence of Pv infection over time. Pv incidence rate (Pv IR) was selected as the measure of frequency to account for the different lengths of person follow-up between the ARC trials. Pv IR was calculated as the number of Pv episodes among any patient over the number of days until a Pv episode or the end of follow-up. Pv incidence rates were analyzed by ARC study, study treatment, and clinical variables of interest. Patients who did not complete study follow-up were not included in the analysis.

A multivariate analysis was conducted to estimate the unbiased association of gametocytemia on admission and post-treatment vivax infection. Covariates for the model were selected based on the univariate analysis and through prior knowledge regarding the association between gametocytemia and Pv relapse. A Poisson model with robust standard errors was constructed and the selection of final covariates was determined through a backwards elimination process to capture joint effects. Confounding was assessed by examining the change in estimate for the exposure when each covariate was individually removed. If the removal of a covariate changed the estimate by at least 10% it was considered an important confounder and the variable was retained. Covariates that were examined included gametocyte carriage at admit, study (ARC1 vs. ARC2), presence of anemia, age, male gender, parasite density, history of malaria, parasite clearance time, fever clearance time, white blood cell count, and duration of symptoms.

### Ethics

Ethics approval for this study was obtained from the ethics committees of the Walter Reed Army Institute of Research (Silver Spring, USA) and the National Ethics Committee for Health Research (Phnom Penh, Cambodia).

## Results

Between October 2006–February 2007 and August 2008–July 2009, 111 and 143 patients with *P. falciparum* malaria were enrolled in the ARC1 and ARC2 trials, respectively. Four and 7 patients were withdrawn or lost to follow-up in the respective studies, leaving a total of 243 patients who completed 28 or 42-day follow-up that were included in the analysis.

Clinical characteristics of the two study populations differed in several ways (Table 1). Due to the minimum 1,000 parasites/µl inclusion criteria in the later study, patients in ARC2 had significantly higher baseline parasitemias, with a median parasitemia double that in ARC1 (12,748 vs. 6,562 parasites/µl). Patients in ARC2 were more likely to be male (79% vs. 64%), less frequently reported a history of malaria in the previous 12 months (33% vs. 41%), and demonstrated slower parasite and fever clearance times (median 78 vs. 64 hours and 16 vs. 9 hours, respectively) than patients in ARC1.

**Table 1:** Clinical characteristics of patients by ARC study.

	ARC1 2006–7 (n = 107)	ARC2 2008–9 (n = 136)	p-value
Age	28 (20–41)	25 (20–35)	0.04
Male gender	68 (64%)	107 (79%)	0.01
History of malaria in previous 12 mos	55 (41%)	45 (33%)	0.01
Duration of symptoms (days)	3 (3–5)	3 (3–4)	0.07
Baseline parasitemia (/µl)	6,562 (2,339–19,849)	12,748 (5,090–34,730)	<0.001*
Baseline parasitemia group			
0–10 K	68 (64%)	60 (44%)	
10 K–100 K	39 (36%)	69 (51%)	
>100 K	0 (0%)	7 (5%)	
Baseline temperature (°C)	38.0 (37.2–38.8)	38.1 (37.4–38.9)	0.24
Fever at admission (T>37.9°C)	58 (54%)	77 (57%)	0.71
Baseline hematocrit (%)	38 (33–41)	39 (37–43)	0.01
Presence of Pf gametocytes on admit	23 (22%)	20 (15%)	0.17
Presence of Pf gametocytes on day 14	16 (15%)	12 (9%)	0.14
Presence of Pf gametocytes anytime during follow-up	40 (37%)	36 (26%)	0.07
Parasite clearance time (hrs)	64 (43–91)	78 (60–90)	0.01
Fever clearance time	9 (0–29)	16 (7–29)	0.01
Developed Pv parasitemia during follow-up**	32 (30%)	27 (20%)	0.07
Developed Pv parasitemia by day 28 of follow-up	32 (30%)	15 (11%)	<0.001
Developed Pf recurrence	4 (3.8%)	8 (5.9%)	0.44

Values are reported as median and interquartile ranges unless otherwise specified.

*The difference in baseline parasitemia reflects a difference in the inclusion criteria between the two trials, with no minimum parasitemia required in ARC1.

**Follow-up was for 28 days and 42 days for ARC1 and ARC2, respectively.

### Gametocyte carriage

Falciparum gametocyte carriage at admission was similar in both studies (Table 1). In the combined analysis, 43/243 (18%) of patients had falciparum gametocytes detected by microscopy at baseline. By day 14, this number had decreased to 28/243 (12%) of patients, with the majority (208/243, 86%) having been treated with 7 days of artesunate. Nearly one-third of patients (76/243 or 31%) had gametocytes detectable by microscopy at some point during follow-up.

**Figure 1:**
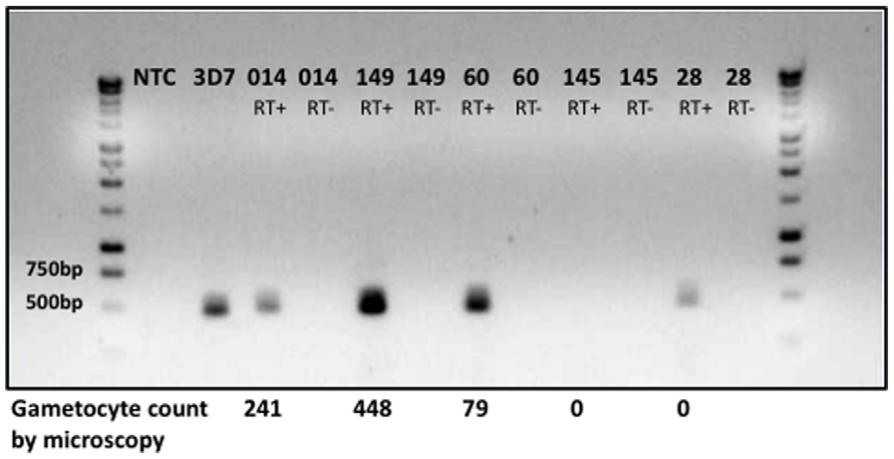
Detection of baseline falciparum gametocytes by RT-PCR of Pfs25. Gel electrophoresis of Pfs25 transcripts by reverse-transcriptase PCR from selected patient samples. Patients 014, 149, and 60 were gametocyte-positive by both microscopy and RT-PCR. Patient 145 was negative by both methods. Patient 28 had subpatent gametocytemia detected by PCR only. 3D7 positive control consisted of 1 ng/µl of gDNA purified from 3D7 Pf erythrocyte culture. Reverse transcriptase-negative controls were done in parallel to ensure there was no DNA contamination of the purified RNA.

Molecular detection of Pf gametocytes by RT-PCR for those patients enrolled in ARC2 showed good concordance with microscopy (Table S1, Figure 1). All but one patient found to be gametocytemic at presentation by microscopy were confirmed by RT-PCR. Additionally, 10 microscopy-negative patients were positive by RT-PCR. Thus, while 15% (20/136) of ARC2 patients had smear-detectable gametocytes at baseline, an additional 6% had subpatent gametocytes identified by RT-PCR.

In the combined data, risk factors associated with baseline patent gametocytemia by univariate analysis included longer duration of symptoms, a greater number of malaria episodes in the previous 12 months, lower baseline hematocrit, lower baseline temperature, and a higher baseline platelet count (Table S2). Gametocyte carriage at presentation was also associated with a faster fever clearance time (median of 5 vs. 18 hrs for those with vs. without gametocytes).

### Post-treatment *P. vivax* infection

**Figure 2:**
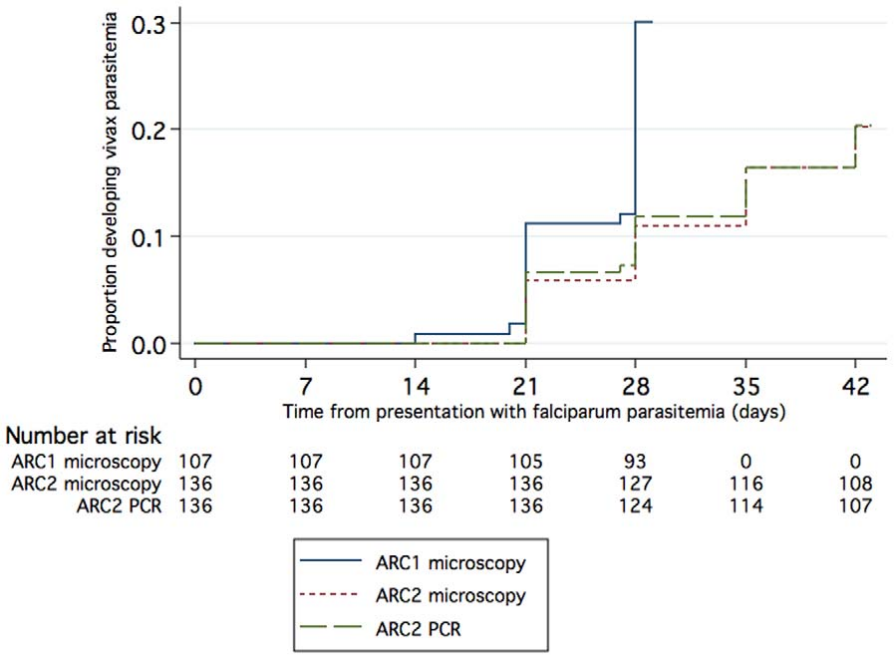
Cumulative proportion of patients with vivax malaria during follow-up based on smear or PCR diagnosis.

59/243 (24%) patients developed *P. vivax* infection during follow-up, 32 by Day 28 in ARC1, and 27 by Day 42 in ARC2 (Table 2, Figure 2). Of these, 20/59 (34%) were febrile or otherwise symptomatic at the time of diagnosis, and 24/59 (41%) had associated Pv gametocytemia. The median time to the identification of *P. vivax* was 28 days in both studies. 55 out of the 59 post-treatment Pv patients had admission blood samples available for 18 s rRNA PCR analysis. Only 9.1% of these (5/55) had detectable vivax parasitemia by PCR at Day 0, suggesting that the majority did not have mixed Pf/Pv blood-stage infection at presentation that was missed by microscopy (Table 2). When PCR instead of microscopy was used to identify post-treatment Pv episodes in the ARC2 patients, the cumulative incidence of Pv parasitemia did not change, though there were discrepant results in 8/136 patients (Figure 2, Table S3).

**Table 2:** Incidence of *P. vivax* infection following treatment.

	No.	Post-treatment *P. vivax*	Incidence rate Pv/1000 over 30 days of follow-up	*P. vivax* detected at Day 0 by PCR	Median day of onset of *P. vivax* following treatment (range)
**ARC1 2006–7**	**107**	**32**	**330.7**	**3/28** * **(11%)**	**28 (14–28)**
Artesunate	72	21	326.4	2	24 (14–28)
Quinine/tetracycline	35	11	339.2	1	28 (21–28)
**ARC2 2008–9**					
Artesunate**	**136**	**27**	**147.8**	**2/27 (7.4%)**	**28 (21–42)**
AS2	72	12	122.7	1	32 (21–42)
AS4	38	7	133.3	0	35 (21–42)
AS6	26	8	246.7	1	25(21–42)
**Total combined**	**243**	**59**	**211.1**	**5/55 (9.1%)**	**28 (14–42)**

*4 Day 0 samples were not available.

**Artesunate was dosed at 2 vs. 4 vs. 6 mg/kg/day×7 days.

The vivax incidence rate (IR) was calculated to account for the different durations of follow-up between the two studies. In ARC1, Pv IR was 331 per 1000 patient months of follow-up. This was more than double the ARC2 Pv IR of 148 per 1000 patient months of follow-up (Table 2). Just comparing Day 28 Pv infection rates, 30% (32/107) of patients in ARC1 vs. 11% (27/136) of patients in ARC2 developed Pv by Day 28 (p<0.001) (Table 1). Incident rates were similar between the artesunate and quinine/tetracycline groups in ARC1.

### Risk Factors for post-treatment *P. vivax* infection

On combined univariate analysis, the most prominent risk factors for the development of post-treatment vivax infection were the presence of falciparum gametocytes on peripheral smear and a shorter fever clearance time (Table 3). While the greatest risk was found in those with baseline Pf gametocytemia by RT-PCR, gametocytes present at Day 14 or anytime during follow-up were also associated with a higher rate of post-treatment Pv infection.

**Table 3:** Comparison of patients with and without *P. vivax* infection by Day 28 post-treatment.

	*P. falciparum* patients with *P. vivax* within 28 days post-treatment (n = 47)	*P. falciparum* patients without P. vivax (n = 196)	p-value
Age	27 (22–34)	26 (20–40)	0.52
Male gender	39 (83%)	136 (69%)	0.06
History of malaria in previous 12 mos	26 (55%)	74 (38%)	0.03
Duration of symptoms (days)	3 (3–5)	3 (2–4)	0.16
Baseline parasitemia (/µl)	7,003 (2,903–18,590)	10,093 (4,090–31,770)	0.04
Baseline temperature (°C)	38.0 (37.5–39.0)	38.0 (37.3–38.9)	0.94
Fever at admission (T>37.9°C)	28 (60%)	107 (55%)	0.54
Baseline hematocrit (%)	38 (35–41)	39 (35–42)	0.11
Presence of anemia (Hct<30%)	2 (4.3%)	12 (6.1%)	1.0
Baseline white blood cell count (×10^3^/µl)	6.5 (4.8–7.7)	5.9 (4.9–7.3)	0.07
Baseline platelet count (×10^3^/µl)	136 (102–166)	97 (69–135)	0.02
Baseline ALT (alanine aminotransferase U/L)*	18 (14–29)	22 (14–33)	0.42
Presence of Pf gametocytes on admit	20 (43%)	23 (12%)	<0.001
Presence of Pf gametocytes on admit by RT-PCR*	10 (67%)	19 (16%)	<0.001
Presence of Pf gametocytes on day 14	12 (26%)	16 (8.2%)	0.001
Presence of Pf gametocytes anytime during follow up	30 (64%)	46 (23%)	<0.001
Parasite clearance time (hrs)	66 (51–90)	72 (52–90)	0.28
Fever clearance time (hrs)	5 (0–23)	15 (5–29)	0.001
Bioassay positive*,**	0	8 (4.1%)	0.60

Values are reported as median and interquartile ranges unless otherwise specified.

*Data only available for ARC2 patients (Pv n = 15, no Pv n = 123).

**Antimalarial activity of sera against *P. falciparum* lab strains in a previously published ex-vivo bioassay, used as a surrogate measure of prior use of antimalarial drugs [15].

In total, of the 43/243 (18%) patients who carried smear-detectable falciparum gametocytes at admission, nearly half (20/43, 47%) had a malaria relapse with *P. vivax* within 28 days compared to 27/200 (14%) patients without admission Pf gametocytemia who also relapsed. Those with smear-detectable baseline gametocytemia demonstrated a 3.5-fold increased incidence rate of vivax malaria post-treatment (IRR = 3.5, 95% CI 2.0–6.0, p<0.001) (Table 4). If used as a diagnostic test, the presence of gametocytes predicted subsequent 28-day Pv infection with a sensitivity of 47% and a specificity of 87% in our population. Similar results were also found when the patients with PCR-detectable *P. vivax* at admission (n = 16) or no baseline samples available for analysis (n = 14) were excluded (IRR = 3.7, 95% CI 2.0–6.8, p<0.001), as well as when Pv parasitemia by PCR was used as the marker of relapse rather than smear positivity. Finally, the association remained statistically significant regardless of trial and drug treatment allocation (data not shown).

**Table 4:** Comparison of post-treatment vivax incidence rate by risk factor.

	Risk present					Risk absent							
	*P. vivax* relapse		No *P. vivax* relapse		Incidence rate/1000/30days	*P. vivax* relapse		No *P. vivax* relapse		Incidence rate/1000/30days			
Risk factor	no.	days of follow-up	no.	days of follow-up		no.	days of follow-up	no.	days of follow-up		Incidence Rate Ratio	95%CI	p value
Male gender	50	1378	125	4662	248	9	244	59	2016	119	2.08	1.01, 4.81	0.03
History of malaria in previous 12 mos	32	881	68	2338	298	27	741	115	4298	161	1.86	1.08, 3.22	0.02
Duration of symptoms >3 days	25	728	55	1946	280	34	894	129	4732	181	1.55	0.88, 2.67	0.10
Baseline parasitemia (/µl) <10 K	35	937	93	3248	251	24	685	91	3430	175	1.43	0.83, 2.52	0.17
Baseline parasitemia (/µl) >50 K	3	77	24	952	87	56	1545	160	5726	231	0.38	0.08, 1.16	0.07
Fever (T>37.9°C)	31	804	104	3822	201	28	818	80	2856	229	0.88	0.51, 1.52	0.62
Anemia (Hct<30%)	4	133	10	336	256	55	1489	174	6324	211	1.21	0.32, 3.29	0.67
Baseline platelet count <150 K	14	413	80	3360	111	10	294	18	756	286	0.39	0.16, 0.98	0.03
Abnormal ALT (>40 U/L)	3	70	19	798	104	24	749	88	3696	162	0.64	0.12, 2.11	0.49
Gametocytes on admit	23	609	20	686	533	36	1013	164	5992	154	3.46	1.96, 5.99	<0.001
Gametocytes on admit by PCR*	16	469	13	546	473	11	350	96	4032	75	6.28	2.74, 15.0	<0.001
Gametocytes on day 14	14	364	13	420	536	44	1223	171	6258	176	3.04	1.54, 5.64	0.001
Gametocytes anytime during follow up	39	1071	37	1260	502	20	551	147	5418	101	4.99	2.84, 9.04	<0.001
Parasite clearance time ≥72 hrs	25	707	100	3766	168	34	915	84	2912	267	0.63	0.36, 1.09	0.08
Fever clearance time >24 hrs	14	378	74	2716	136	45	1244	109	3934	261	0.52	0.26, 0.97	0.03
Bioassay positive*,**	2	77	6	252	182	25	742	103	4326	148	1.23	0.14, 4.94	0.72

*Data only available for ARC2 patients (Pv n = 27, no Pv n = 109).

**Antimalarial activity of sera against *P. falciparum* lab strains in a previously published ex-vivo bioassay, used as a surrogate measure of prior use of antimalarial drugs [15].

Other variables associated with post-treatment vivax infection in the univariate analysis included a history of malaria in the previous 12 months, male gender, a lower baseline parasitemia, and a higher baseline platelet count.

In the multivariate analysis, each of the major risk factors identified in univariate analysis as well as other potential confounding variables, including those that could lead to greater gametocyte carriage, were adjusted for as covariates. After removing the bias from these confounding factors, baseline gametocytemia was associated with a 3.0-fold increased rate of relapse with vivax malaria (IRR = 3.0, 95% CI 1.9–4.7, p<0.001) (Table 5). Male gender and enrollment in ARC1 were the other identified independent risk factors for relapse.

**Table 5:** Attributable risk of vivax infection post-treatment after adjusting for covariates*.

Risk factor	Incidence rate ratio	95%CI		p-value
Gametocytes on admit	2.95	1.85	4.69	<0.001
Enrollment in ARC1	2.10	1.30	3.20	0.002
Male gender	2.41	1.27	4.57	0.007
History of malaria in previous 12 mos	1.46	0.95	2.26	0.074
Fever clearance time >24 hrs	0.70	0.40	1.22	0.434

*Also accounting for confounding from age, parasite density, parasite clearance time, WBC count, history of malaria, and presence of anemia.

## Discussion

This analysis uses clinical, microscopic, and molecular data from two malaria treatment trials conducted in an area co-endemic for *P. falciparum* and *P. vivax* to show that falciparum gametocytes seen at presentation in patients with *P. falciparum* malaria is associated with subsequent relapse with *P. vivax* malaria. The finding is robust despite differences in the two study populations, including a higher Pv incidence rate in ARC1 which may reflect changing malaria epidemiology in this region. When baseline subpatent gametocytemia was detected using Pfs25 RT-PCR, the association was strengthened. Gametocyte carriage continued to be an independent risk factor even after controlling for potential confounding variables related to exposure and immunity. Prior inadequate drug treatment is another potential confounder that may have contributed to high gametocyte carriage rates in those who relapsed. However, antimalarial use in the previous 30 days was an exclusion criterion in both studies, and an ex vivo anti *P. falciparum* bioassay, conducted on all baseline ARC2 samples, failed to detect anti-falciparum activity in plasma collected from any of the patients who developed post-treatment vivax infection [15].

The low proportion of patients with PCR-detectable Pv at baseline and the excellent efficacy of the study drugs against Pv blood stages [3] suggest that most of the post-treatment vivax infections detected in our study represent reactivation of dormant hypnozoites rather than recrudescent blood stage Pv parasites not detected at the time of diagnosis. In addition, post-treatment vivax infections are unlikely to have resulted from newly inoculated Pv infections. This is supported by the relatively short follow-up periods and a relatively low entomological inoculation rate in this region. Furthermore, patients enrolled in ARC1 stayed in a structure adjacent to the study ward in the town of Tasanh, a largely transmission-free area, for 21 of the 28 days of follow-up.

Taken together, these findings suggest that falciparum gametocytes on a blood smear at presentation may be a marker for liver-stage *P. vivax* infection in patients with occult mixed infection. We offer two possible explanations for why this might be the case. First, the presence of Pf gametocytes may indicate increased malaria exposure in general and resultant higher levels of acquired immunity. In this case, patients carrying gametocytes may be more likely to have acquired a Pv infection in the past and to harbor hypnozoites in their liver when they are infected with Pf. The theory that repeated exposure may lead to heightened acquired immunity and subsequent gametocytemia is supported by previous *in vitro* experiments whereby falciparum parasites in culture displayed increased levels of gametocytogenesis after exposure to immune Pf sera or anti-Pf antibodies [16]. In our analysis, gametocytemia was associated with a greater number of previous episodes of malaria and a faster fever clearance time. Similarly, male gender was another independent risk factor for Pv relapse, and men working on the forest fringe in Cambodia are thought to have the greatest exposure to malaria. However, prior immunity and exposure do not seem to completely explain the association, as other plausible markers of immunity, such as a history of malaria and shorter fever clearance time, were not independently related to Pv relapse. Gametocytemia remained an important factor even after controlling for these variables as well as after controlling for male gender, duration of symptoms, presence of anemia, and baseline parasite density.

A second explanation is that competition from a second malaria species may boost falciparum gametocytogenesis as an evolutionary adaptation. In this scenario, competition from a second co-circulating species or immune stress induced by the presence of another species may induce falciparum parasites to divert from the asexual to the sexual life cycle, accelerating transmission to new hosts. This theory is supported by observations from mixed *P. falciparum*/*P. malariae* infections. In a review of Boyd's early malariotherapy studies in which neurosyphilis patients were experimentally inoculated with malaria sporozoites, McKenzie et al. found that those inoculated with *P. falciparum* following *P. malariae* inoculation developed 3–4 fold higher falciparum gametocyte densities and gametocytes that appeared 4 days earlier than in their counterparts with single species *P. falciparum* infection [17]. Similarly, in a drug treatment trial (SP+AQ) involving 128 children (aged 6 months to 10 years) in Kenya, the group of 21 children with mixed *P. falciparum*/*P. malariae* infection demonstrated higher gametocyte densities over 28 days with an area under the curve (AUC) more than twice that of those with *P. falciparum* mono-infection [18].

The natural history of mixed infections is characterized by a pattern of alternating dominance of the two species, where often only one species is patent despite an underlying mixed infection. This pattern was evident when Boyd simultaneously inoculated two malaria-naïve patients with *P. falciparum* and *P. vivax* sporozoites and followed daily blood smears [19], [20]. It has also been seen more recently in the field setting among asymptomatic semi-immune children in Papua New Guinea [21]. In light of these observations, we hypothesize that, prior to presentation, patients who went on to develop Pv were originally inoculated with a mixed Pf/Pv infection in which co-circulating Pv parasites induced gametocytogenesis in their Pf counterparts. These blood-stage Pv parasites may have subsequently been “outcompeted” by the dominant Pf infection such that by the time the patient presented with symptomatic asexual Pf parasitemia, the only evidence of prior Pv infection were hypnozoites in the liver and circulating falciparum gametocytes.

Alternatively, these results could lead to the conclusion that falciparum gametocytes directly trigger reactivation of vivax hypnozoites in the liver. However, in the Myanmar ACT study, a single dose of primaquine, which was very effective at decreasing post-treatment gametocyte carriage, did not seem to have an effect on the post-treatment Pv incidence rate [8]. Similarly, while our artesunate-treated patients had lower rates of post-treatment gametocyte carriage than those treated with quinine plus tetracycline, the two treatment groups relapsed with vivax malaria at a similar rate. We also did not find any relationship between gametocyte density and the risk of 28-day relapse, or timing of gametocyte appearance and time to Pv relapse.

A recent study by Douglas et al., based on 15 years of clinical trial data from the Thai-Burmese border, reports a similar association in falciparum-infected patients who relapsed with vivax malaria. Though the reported effect size was smaller (AHR 1.4, 95%CI 1.1–1.7), those with Pf gametocytemia at enrollment still represented the single group with the highest risk of Pv recurrence (41% by day 63) according to baseline risk factors [9]. Confirmatory findings should be sought in other regions with mixed species *Plasmodium* infections as well.

Our study adds to the literature by providing insights into the pathophysiology of relapse in patients with occult Pf/Pv infection in southeast Asia and identifying an easily distinguished marker for patients with falciparum malaria who are at risk for vivax relapse. Recognizing the high likelihood of occult *P. vivax* coinfection in gametocytemic individuals further highlights the potential public health gains that could be achieved with more widespread G6PD testing [22]. In areas where G6PD testing is currently available, our results suggest that treating gametocytemic patients with a full course of primaquine may reduce the proportion of patients with vivax relapse. Such an approach could stratify the risk of G6PD related hemolysis, reserving presumptive full course primaquine treatment for those at a higher risk of relapse. In our study area, adopting such a strategy would target ∼18% of patients while potentially preventing up to 39%, i.e. the population attributable fraction, of early vivax relapses. In Thailand, falciparum-infected patients are already given one dose of primaquine with an ACT to target gametocytes as part of a transmission-blocking strategy. In the midst of malaria elimination efforts on the Thai-Cambodian border, failure to offer a full course of primaquine to falciparum patients with gametocytes could represent a missed opportunity to also reduce rates of *P. vivax* malaria, arguably the harder species to eliminate because of its propensity for relapse.

In summary, in Cambodian patients with acute falciparum malaria, the presence of *P. falciparum* gametocytes on blood smear at presentation is associated with subsequent *P. vivax* relapse; this has important implications for malaria treatment and control strategies in settings where mixed *P. falciparum/P. vivax* infections are common.

## Supporting Information

Table S1 **Comparison of microscopy and PCR detection of falaciparum gametocytes at baseline in ARC2 patients.**
(TIFF)

Table S2 **Risk Factors for gametocyte carriage at admit.** Values are reported as median and interquartile ranges unless otherwise specified. *Data only available for ARC2 patients (Pfg n = 20, no Pfg n = 116). **Antimalarial activity of sera against *P. falciparum* lab strains in a previously published ex-vivo bioassay, used as a surrogate measure of prior use of antimalarial drugs [15].
(TIFF)

Table S3 **Comparison of microscopy and PCR detection of post-treatment Pv parasitemia in ARC2 patients.**
(TIFF)
