# Supplementary figures and images for: Plasmodium falciparum Gametocyte Carriage Is Associated with Subsequent Plasmodium vivax Relapse after Treatment
Source: PLoS One. 2011 Apr 20;6(4):e18716. doi: 10.1371/journal.pone.0018716 (PMC3080384; doi:10.1371/journal.pone.0018716)

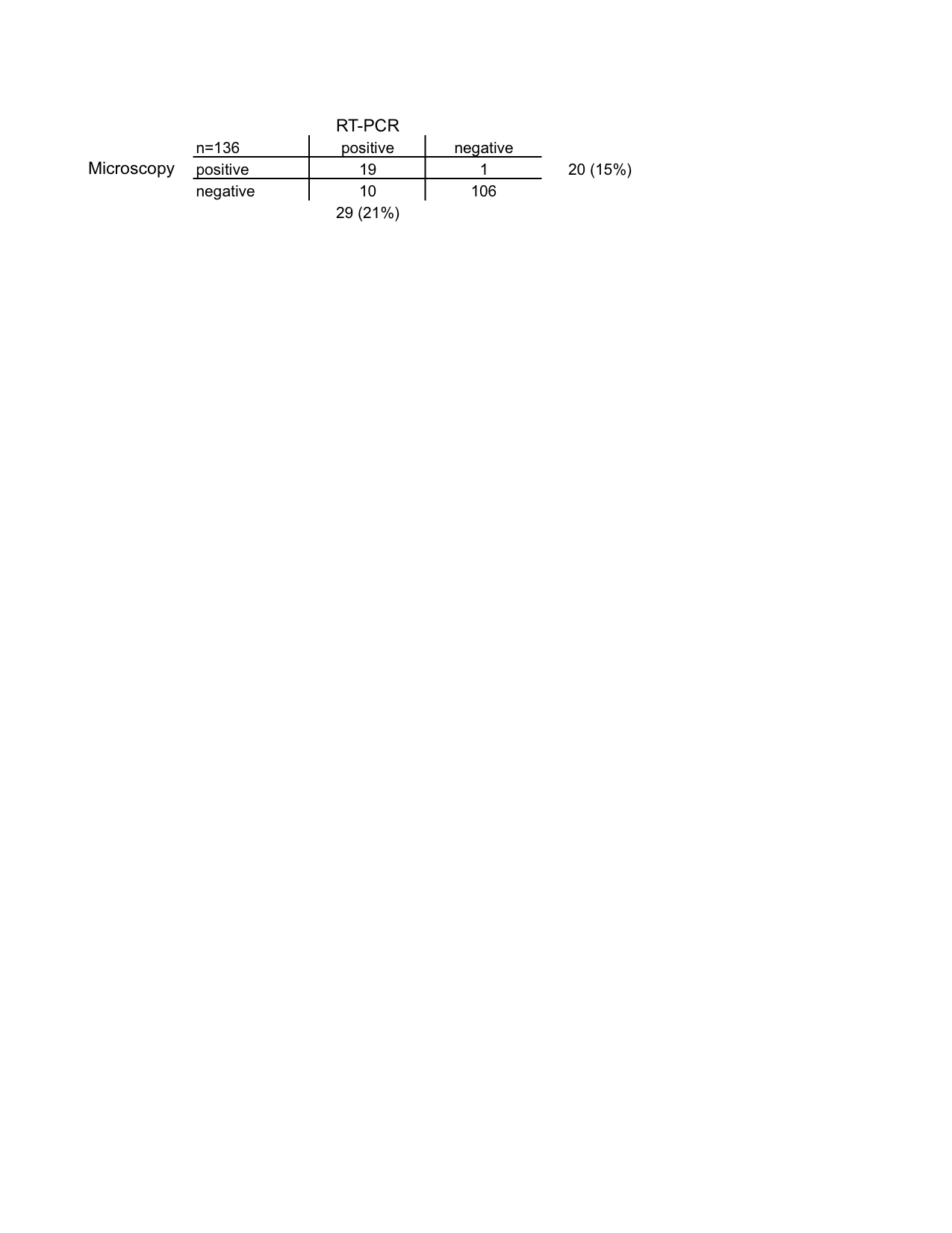

Supplement: Table S1 — Comparison of microscopy and PCR detection of falaciparum gametocytes at baseline in ARC2 patients. (TIFF) [file pone.0018716.s001.tif]

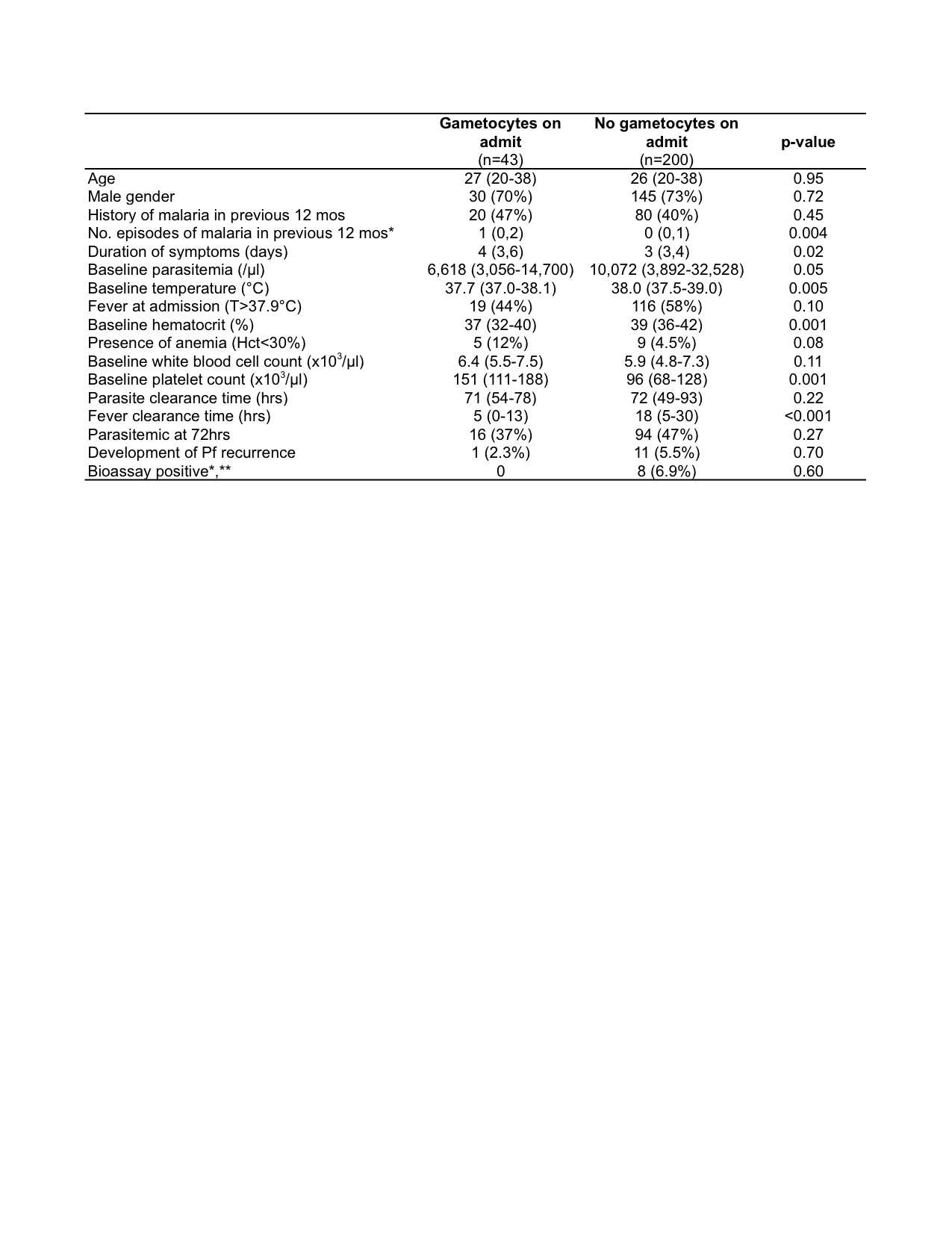

Supplement: Table S2 — Risk Factors for gametocyte carriage at admit. Values are reported as median and interquartile ranges unless otherwise specified. *Data only available for ARC2 patients (Pfg n = 20, no Pfg n = 116). **Antimalarial activity of sera against P. falciparum lab strains in a previously published ex-vivo bioassay, used as a surrogate measure of prior use of antimalarial drugs [15]. (TIFF) [file pone.0018716.s002.tif]

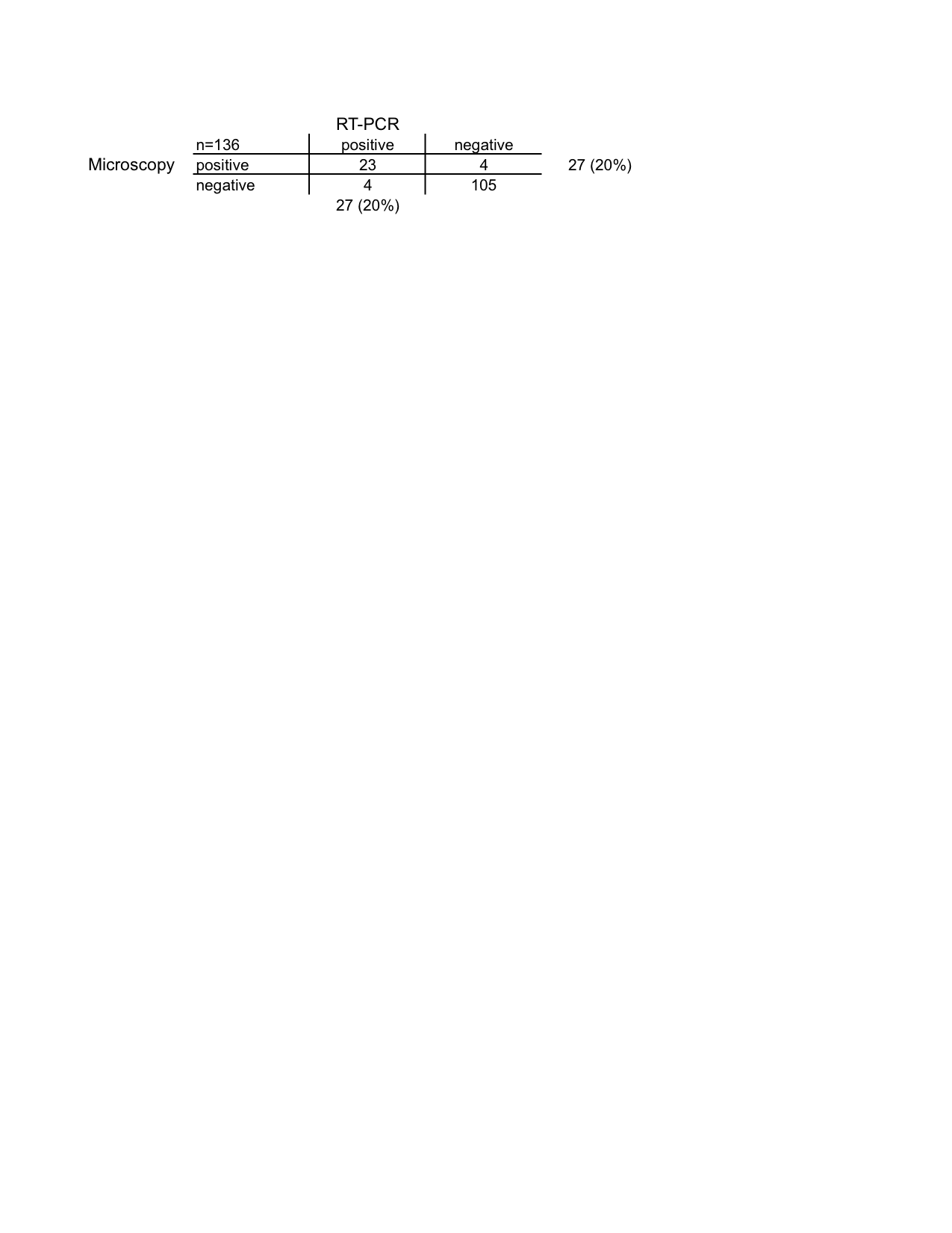

Supplement: Table S3 — Comparison of microscopy and PCR detection of post-treatment Pv parasitemia in ARC2 patients. (TIFF) [file pone.0018716.s003.tif]
